# Operative diagnosis for revision total hip arthroplasty is associated with patient-reported outcomes (PROs)

**DOI:** 10.1186/1471-2474-14-210

**Published:** 2013-07-17

**Authors:** Jasvinder A Singh, David G Lewallen

**Affiliations:** 1 Medicine Service and Center for Surgical Medical Acute care Research and Transitions (C-SMART), Birmingham VA Medical Center, Birmingham, AL, USA; 2 Department of Medicine at School of Medicine, University of Alabama, at Birmingham, Birmingham, AL, USA; 3 Division of Epidemiology at School of Public Health, University of Alabama, at Birmingham, Birmingham, AL, USA; 4 Department of Orthopedic Surgery, Mayo Clinic College of Medicine, Rochester, MN, USA; 5 Birmingham VA Medical Center and University of Alabama at Birmingham, Birmingham, AL, USA

**Keywords:** Total hip replacement, Arthroplasty, Joint replacement, Patient-reported outcomes, Diagnosis

## Abstract

**Background:**

Little is known about the impact of the reason for revision total hip arthroplasty (THA) on the outcomes following revision THA. In this study, our objective was to assess the association of operative diagnosis with patient-reported outcomes (PROs) after revision THA.

**Methods:**

We used prospectively collected data from the Mayo Clinic Total Joint Registry that collects pre- and post-operative pain and function outcomes using a validated Hip questionnaire, on all revision THAs from 1993–2005. We used logistic regression to assess the odds of moderate-severe index hip pain and moderate-severe limitation in activities of daily living (ADLs) 2- and 5-years after revision THA. We calculated odds ratios (OR) and 95% confidence intervals (CIs).

**Results:**

For the 2- and 5-year cohorts, the operative diagnosis was loosening/wear/osteolysis in 73% and 75%; dislocation/bone or prosthesis fracture/instability or non-union in 17% and 15%; and failed prior arthroplasty with components removed/infection in 11% and 11%, respectively. In multivariable-adjusted analyses that included preoperative ADL limitations, compared to patients with loosening/wear/osteolysis, patients with dislocation/fracture/instability/non-union had OR of 2.2 (95% CI, 1.3-3.5; p = 0.002) for overall moderate-severe ADL limitation and those with failed prior arthroplasty/infection had OR of 1.6 (95% CI, 1.0-2.8; p = 0.06). At 5-years, ORs were lower and differences were no longer significant. Moderate-severe pain did not differ significantly by diagnosis, at 2- or 5-years in multivariable adjusted analyses, with one exception, i.e. failed prior arthroplasty/infection had a trend towards significance with odds ratio of 1.9 (95% CI, 0.9-3.8; p = 0.07).

**Conclusions:**

Operative diagnosis is independently associated with ADL limitations, but not pain, at 2-years after revision THA. Patients should be informed of the risk of poorer short-term outcomes based on their diagnosis.

## Background

Revision total hip arthroplasty (THA) is a common procedure performed for improvement in pain and function in patients who have previously undergone primary THA [1]. Among arthroplasty procedures, revision THA is a common procedure. The annual volume of revision THA is increasing rapidly [2,3]. Based on the National Inpatient Sample (NIS) sample, compared to 40,800 revision THAs performed in in 2005, it is projected that the volume will increase by 137% to 96,700 annually by year 2030 [3].

The increase in the volume of revision THA at 137% is not too dissimilar to that of primary THA projected to increase by 174% in the same period [3]. On the other hand, most published literature has focused on predictors of outcomes in patients with primary THA, with very few studies focusing on revision THA. One important potential predictor of outcomes is the operative diagnosis for revision THA, but even fewer studies have examined this association. In a previous study, patients undergoing revision for loosening had better functional outcome (WOMAC function score) at 1–2 year follow-up compared to other diagnoses (infection, instability or fracture) [4]. A recent study found that functional improvements were lower in patients with infection compared to patients with mechanical or pain causes for revision hip arthroplasty [5]. Other studies of predictors of outcomes after revision THA have reported the following variables to be associated: better preoperative pain scores and fewer comorbidities with functional outcomes [6]; higher comorbidity with major complications [7]; younger age, obesity and depression [8], higher body mass index (BMI) [9], and female gender with worse pain outcomes [4,8,10]; and higher BMI and worse preoperative scores with worse composite pain and function outcome [11].

The objective of this study was to examine whether the operative diagnosis was associated with pain and function outcomes in patients undergoing revision THA. We hypothesized that the (1) operative diagnosis of loosening, wear or osteolysis will be associated with better pain and function outcomes at both 2- and 5-years post-revision THA compared to other diagnoses and (2) that the association will be attenuated by adjustment for preoperative status and other covariates.

## Methods

We describe the methods and results as recommended in the Strengthening of Reporting in Observational studies in Epidemiology (STROBE) statement [12].

### Setting and participants

In this observational cohort study, we used prospectively collected data from the Mayo Clinic Total Joint Registry, a large U.S. institutional registry that collects data on every patient who undergoes hip arthroplasty at the Mayo Clinic, Rochester [13,14]. At 2- and 5-years validated pain and function surveys are administered to patients at the clinic visit, by mail or on the telephone, by trained, registry staff. The Mayo Hip questionnaire has been validated [13-15]. Several papers using these data have been published [8,16]. Patients were included in this study if they underwent revision THA between 1993 and 2005 and completed either a 2- or 5-year patient survey. The study was approved by the Mayo Clinic Institutional Review Board.

### Outcomes of interest

We used the Mayo Hip questionnaire, a validated instrument [13-15], as the source for both PROs of interest, overall moderate-severe ADL limitation and moderate-severe pain 2- or 5-years after revision THA. We categorized the responses to limitations in seven activities including walking, climbing stairs, putting on shoes/socks, picking up objects from the floor, sitting, getting in/out of the car, rising from chair into ‘no’ , ‘mild’ , ‘moderate’ or ‘severe’ limitation for each activity. Presence of ≥3 activities with moderate or severe limitation was classified as overall moderate-severe ADL limitation (reference, no/mild ADL limitation), as previously [8,17]. The pain question, similar to the pain question in Harris Hip Score, a validated THA outcome instrument [18-20], stated “How much pain do you have in your operated hip?” Patients could respond- ‘none’ , ‘mild’ , ‘moderate’ or ‘severe’. None/mild pain was the reference category and moderate and severe categories were combined into moderate-severe pain, based on an a priori decision before data analyses, as in previous studies [8,16]. These *a priori* decisions were based on recommendations from an experienced orthopedic surgeon (D.G.L.), who viewed moderate-severe ADL limitation or pain as undesirable outcomes of arthroplasty.

### Predictor of interest

Operative diagnosis was the main predictor of interest. Based on *a priori* decision, diagnoses were lumped into 3 categories, as previously [8,21-23]: (1) loosening, wear or osteolysis; (2) dislocation, bone or prosthesis fracture, instability or non-union; and (3) failed prior arthroplasty with components removed or infection.

### Covariates of interest

Since several clinical, demographic and implant related factors have been previously shown to be associated with outcomes after THA, they were included in the analyses as covariates and potential confounders [8,11,16]. These included: (1) demographic factors- age and gender; (2) clinical factors - body mass index (BMI) [24], American Society of Anesthesiologists (ASA) class [25,26], depression, anxiety and medical comorbidity which was assessed by Deyo-Charlson index, a validated comorbidity measure [27], consisting of 17 comorbidities [28,29], based on the presence of International Classification of Diseases- ninth revision (ICD)-9 codes; (3) distance from the medical center; and (4) preoperative limitation in 7 ADLs or preoperative pain, in the respective model. As previously, age was categorized into ≤60, 61–70, 71–80 and >80, BMI into ≤25, 25.1-29.9, 30–34.9, 35–39.9 and ≥40, ASA class into I-II vs. III-IV [8,16] and distance from the medical center into 0–100 miles, >100-500 miles, >500 miles [24,30,31]. Depression and anxiety were assessed by the presence of ICD-9 codes in medical records before the THA.

### Data sources

Data on the dates of the THA, demographic details (age, gender), BMI, ASA class, operative diagnosis, distance from the medical center and preoperative limitation in 7 ADLs and preoperative pain were obtained from the Mayo Total Joint Registry, since they are captured for every patient. ICD-9 codes for the Deyo-Charlson comorbidities, anxiety and depression were obtained from linked Mayo Clinic electronic databases.

### Bias

We anticipated non-response at both 2- and 5-years, higher at 5- than 2-years. We minimized confounding bias by including multiple covariates known or suspected to be associated with use of ADL limitation or pain after THA.

### Sample size

Our plan was to assemble a large enough sample to study without having too long a study period, therefore we chose all eligible patients from 1993 to 2005. We did not perform any formal sample size calculations.

### Statistical analyses

We used univariate and multivariable logistic regression models to assess the association of operative diagnosis with the odds of moderate-severe ADL limitation and moderate-severe pain at both 2- and 5-years post-revision THA. Logistic regressions were performed using a generalized estimating equations (GEE) approach to adjust the standard errors for the correlation between observations on the same subject due to both hips having been replaced and/or multiple operations on the same hip. The multivariable model included age, gender, BMI, ASA class, anxiety, depression, Deyo-Charlson index, distance from the medical center and preoperative limitation in 7 activities or preoperative pain (in respective models). Odds ratios (ORs) with 95% confidence intervals (CIs) were calculated. A p-value ≤ 0.05 was considered statistically significant. We decided *a priori* to perform only four multivariable analyses for moderate-severe ADL limitation and moderate-severe pain at 2- and 5-years to avoid multiple comparisons, which would be needed, had we chosen individual ADLs as outcomes. Descriptive univariate analyses of each ADL were examined as exploratory analyses and presented in an Additional file 1. Sensitivity analyses were performed for the ADL limitation outcome (significantly associated), by adjusting the main multivariable-adjusted model additionally for ipsilateral knee involvement (model 1) or for ipsilateral knee involvement and preoperative index hip pain (model 2).

## Results

The 2- and 5-year revision THA cohorts had a mean age of 66 and 65 years, 54% and 54% were female, and 30% and 32% were younger than 60 years of age respectively. BMI was ≥40 kg kg/m^2^ in 7% and 6% and ASA score was class I/II in 52% and 57% respectively (Table 1). The operative diagnosis was loosening, wear or osteolysis in 73% and 75%; dislocation, bone or prosthesis fracture, instability or non-union in 17% and 15%; and failed prior arthroplasty with components removed or infection in 11% and 11%, respectively (Table 1).

**Table 1:** Clinical characteristics of revision THA cohorts

	**Mean ± standard deviation or n (%)**	
	**2-yr FU**	**5-yr FU**
	**(n = 2,687)**	**(n = 1,627)**
Age	66 ± 13	65 ± 13
% female	54%	54%
BMI (kg/m^2^)	28 ± 6	28 ± 6
Age groups		
≤60 yrs	30%	32%
>60-70 yrs	27%	29%
>70-80 yrs	34%	32%
>80 yrs	10%	7%
BMI (kg/m^2^)		
≤24.9	1%	1%
25-29.9	28%	28%
30-34.9	39%	40%
35-39.9	22%	21%
≥40	7%	6%
ASA Score		
Class I	3%	4%
Class II	49%	53%
Class III	47%	43%
Class IV	<1%	<1%
Operative diagnoses		
Loosening/Wear or Osteolysis	73%	75%
Dislocation, Bone or Prosthesis Fracture, Instability, Non-Union	17%	15%
Failed Prior Arthroplasty with Components Removed or Infection	11%	11%
Deyo-Charlson Index	1 ± 2	1 ± 1
Depression	6%	6%
Anxiety	3%	3%

--, not applicable; numbers rounded to the nearest digit and therefore may not always add up to 100%.

Compared to non-responders, responders at 2-years post-revision THA were less likely to have BMI 35–39.9 (compared to BMI < 25), higher Deyo-Charlson index, ASA class III-IV, and operative diagnoses of dislocation/fracture or failed arthroplasty with components removed/infection (Additional file 1). At 5-years, compared to non-responders, post-revision THA responders were less likely to have ASA class III-IV, higher Deyo-Charlson index, and operative diagnoses of dislocation/fracture or failed arthroplasty with components removed/infection.

### Unadjusted analyses: limitations in activities of daily living (ADLs) and pain

At 2-year and 5-years post revision THA, moderate-severe overall ADL limitation was reported by 51% and 53% with loosening/wear/osteolysis, 66% and 65% with dislocation/fracture/instability/non-union, and 68% and 65% with failed prior arthroplasty/infection (Table 2). Moderate-severe pain at 2- and 5-years was reported by 18% and 19%, 18% and 23% and 18% and 23%, respectively (Table 2). Limitation in each ADL is shown in Additional file 1.

**Table 2:** Unadjusted association of diagnosis with moderate-severe ADL limitation and moderate-severe pain after revision THA

	**2-years**				**5-years**			
	**n/N (%)**	**OR**	**95% CI**	**p-value**	**n/N (%)**	**OR**	**95% CI**	**p-value**
**Moderate-severe overall limitations**								
Loosening/Wear or Osteolysis	940/1862 (50.5%)	1.0 (Ref)			616/1158 (53.2%)	1.0 (Ref)		
Dislocation, Bone or Prosthesis Fracture, Instability, Non-Union	279/423 (66%)	**1.9**	**(1.5, 2.4)**	**<0.01**	147/228 (64.5%)	**1.6**	**(1.2, 2.2)**	**<0.01**
Failed Prior Arthroplasty with Components Removed or Infection	185/274 (67.5%)	**2.0**	**(1.6, 2.7)**	**<0.01**	108/166 (65.1%)	**1.6**	**(1.2, 2.3)**	**<0.01**
**Moderate-severe pain**								
Loosening/Wear or Osteolysis	329/1872 (17.6%)	1.0 (Ref)			216/1159 (18.6%)	1.0 (Ref)		
Dislocation, Bone or Prosthesis Fracture, Instability, Non-Union	75/416 (18%)	1.0	(0.8, 1.4)	0.83	51/226 (22.6%)	1.3	(0.9, 1.8)	0.17
Failed Prior Arthroplasty with Components Removed or Infection	47/265 (17.7%)	1.0	(0.7, 1.4)	0.95	38/166 (22.9%)	1.3	(0.9, 1.9)	0.19

*Ref* Reference category, *OR* odds ratio, *95% CI* 95% confidence interval, *OR* odds ratio.

Bold represents significant odds ratio with p–value <0.05.

Compared to patients with loosening/wear/osteolysis, patients in the other two operative diagnoses categories were twice as likely to report moderate-severe overall ADL limitation 2-years after revision THA (Table 2). At 5-years, patients in either of the two diagnostic categories were 1.6-times as likely to report moderate-severe ADL limitation each, respectively. There were no significant differences in moderate-severe pain by diagnostic category at 2- and 5-years after revision THA (Table 2).

### Multivariable-adjusted analyses

In analyses adjusted for 15-additional variables including preoperative limitations in 7 ADLs, compared to patients with loosening/wear/osteolysis, patients with dislocation/fracture/instability/non-union had significantly higher odds (2.2-times; p = 0.002) of moderate-severe ADL limitation at 2-years post-revision THA (Table 3); those with failed prior arthroplasty/infection showed a trend towards significance with odds ratio of 1.6 (p = 0.06). At 5-years, differences were no longer significant for moderate-severe ADL limitation by diagnosis, after multivariable adjustment. As in the unadjusted analyses, moderate-severe pain did not differ significantly by diagnostic category, at 2- or 5-years in multivariable adjusted analyses, with one exception, i.e. failed prior arthroplasty/infection had a trend towards significance with odds ratio of 1.9 (p = 0.07).

**Table 3:** Multivariable-adjusted association of diagnosis with overall moderate-severe limitation and moderate-severe pain 2- and 5-years after revision THA

	**2-years**			**5-years**		
	**OR**	**95% CI**	**p-value**	**OR**	**95% CI**	**p-value**
**Moderate-severe overall ADL limitation**^**a**^						
Loosening, Wear or Osteolysis*	**1.0 (Ref)**			1.0 (Ref)		
Dislocation, Bone or Prosthesis Fracture, Instability, Non-Union	**2.2**	**(1.3, 3.5)**	**0.002**	1.4	(0.8, 2.6)	0.24
Failed Prior Arthroplasty with Components Removed or Infection	1.6	(1.0, 2.8)	0.06	1.1	(0.6, 2.0)	0.79
**Moderate-severe pain**^**b**^						
Loosening, Wear or Osteolysis*	1.0 (Ref)			1.0 (Ref)		
Dislocation, Bone or Prosthesis Fracture, Instability, Non-Union	0.7	(0.4, 1.4)	0.32	1.0	(0.5, 2.1)	0.98
Failed Prior Arthroplasty with Components Removed or Infection	1.2	(0.7, 2.2)	0.48	1.9	(0.9, 3.8)	0.07

*Reference category: Loosening/Wear or Osteolysis.

^a^Main ADL limitation model was adjusted for 15 covariates/confounders: Age, gender, BMI, ASA class, distance from the medical center, anxiety, depression, Deyo-Charlson index, preoperative limitation in 7 activities.

^b^Adjusted for 9 additional covariates/confounders: Age, gender, BMI, Deyo-Charlson comorbidity score, ASA class, distance from the medical center, anxiety, depression and preoperative pain.

Bold represents significant odds ratio with p–value <0.05.

Sensitivity analyses that additionally adjusted for ipsilateral knee involvement (model 1) and ipsilateral knee involvement and preoperative index THA pain severity (model 2) showed that the interpretation or significance level did not change for the associations with moderate-severe ADL limitations at 2-years (Table 4).

**Table 4:** Sensitivity analyses for moderate-severe ADL limitation additionally adjusting the main multivariable-adjusted models

	**2-years**			**5-years**		
	**OR**	**95% CI**	**p-value**	**OR**	**95% CI**	**p-value**
**Model 1: Main ADL limitation model**^**a **^**additionally adjusted for ipsilateral knee involvement**						
Dislocation, Bone or Prosthesis Fracture, Instability, Non-Union	**2.5**	**(1.4, 4.4)**	**0.003**	1.6	(0.7, 3.4)	0.25
Failed Prior Arthroplasty with Components Removed or Infection	1.5	(0.8, 2.9)	0.22	1.4	(0.7, 3.0)	0.39
**Model 2: Main ADL limitation model**^**a **^**additionally adjusted for ipsilateral knee involvement, preoperative pain**						
Dislocation, Bone or Prosthesis Fracture, Instability, Non-Union	**2.3**	**(1.3, 4.3)**	**0.006**	1.7	(0.8, 3.7)	0.17
Failed Prior Arthroplasty with Components Removed or Infection	1.4	(0.7, 2.7)	0.36	1.3	(0.6, 2.9)	0.46

Main Model Adjusted for 15 covariates/confounders: Age, gender, BMI, ASA class, distance from the medical center, anxiety, depression, Deyo-Charlson index, preoperative limitation in 7 activities.

Bold represents significant odds ratio with p–value <0.05.

## Discussion

We found that operative diagnosis for revision THA was associated with overall moderate-severe ADL limitation in univariate analyses at both 2- and 5-years, but not moderate-severe pain. The associations between operative diagnosis and ADL limitations remained significant at 2-years after multivariable adjustment for variables including preoperative limitations. Associations were not significant at 5-years for moderate-severe ADL limitations in multivariable-adjusted analyses. Several findings in this study merit further discussion.

We found that operative diagnoses other than loosening/wear/osteolysis were independently associated with poorer functional outcome, moderate-severe ADL limitation 2-years after revision THA. This is an interesting finding and supports the previous finding from a study of 222 patients that underwent revision THA that reported better functional outcome (WOMAC function score) in patients with aseptic loosening at 1–2 year follow-up compared to other diagnoses including infection, instability or fracture [4]. The previous study adjusted for age, gender, body mass index, Charnley class, pre-operative WOMAC function and pain scores, preoperative SF-12 mental component score and various implant and surgery related factors. Similarly, our study adjusted for a variety of demographic, clinical and health care access factors. Our study had a much larger sample size (10- and 5-times for 2- and 5-year samples respectively), examined pain and function outcomes at both 2- and 5-years and examined three diagnoses categories (compared to two diagnoses categories in previous study). Thus, our study extends the findings from this previous study.

We found that diagnosis of dislocation/fracture/instability/non-union and failed prior arthroplasty/infection were associated with 2- and 1.6-times the risk of moderate-severe ADL limitation 2-years post-revision THA. These findings confirm those findings from a recent study with a small sample size that found that functional improvements were lower in patients with infection compared to patients with mechanical or pain causes for revision hip arthroplasty [5]. Our study extends findings from an inpatient setting to 2-years post-revision THA. Diagnosis other than loosening/wear/osteolysis may lead to a lower compliance with post-THA rehabilitation given a more challenging early postoperative course for these patients and possibly higher rates of muscle atrophy prior to surgery. Since our analyses already adjusted for preoperative limitation, differences in preoperative status don’t explain these findings. Thus, our results extend the findings from the previous study, and add to the current literature.

An interesting observation was that while diagnosis was associated with ADL limitation, it was not significantly associated with moderate-severe pain at 2- or 5-years, in unadjusted or adjusted analyses. This indicates uncoupling of ADL limitation and pain outcomes, indicating different mechanisms for these some what correlated, but different domains and outcomes. This implies that patients undergoing revision THA can expect similar good results for pain, regardless of the opertative diagnosis for undergoing revision THA. One exception was that a diagnosis of failed prior arthroplasty/infection was associated with an odds ratio of 1.9 for moderate-severe pain at 5-years with a trend towards significance (p = 0.07), compared to loosening/wear/osteolysis.

Our findings must be interpreted considering study limitations. Despite our attempt to control for multiple factors, residual confounding is possible, due to cohort study design. It remains to be seen whether our findings from single center are generalizable to other settings; however, clinical and demographic characteristics of our study cohort were similar to other cohorts in the published studies of revision THA. Non-response may have biased our results towards null, meaning that we may have missed some significant findings. The non-response rate was higher at 5-years, which makes those findings potentially more biased.

Our study has several strengths including a large sample size (>5-times most previous studies), analyses of prospectively collected data, examination of both pain and ADL limitations at 2-and 5-years, multivariable-adjusted analyses accounting for multiple covariates and confounders, and several sensitivity analyses that confirmed the robustness of our findings.

## Conclusions

In conclusion, in this study, we found that compared to loosening/wear/osteolysis, operative diagnosis of dislocation/fracture/instability/non-union and failed prior arthroplasty/infection were each associated with higher risk of moderate-severe ADL limitation at 2-years. We did not observe any significant association between operative diagnosis and moderate-severe pain. The findings from our study can be used to better inform the patients about expected pain and function outcomes of revision THA. More work is needed to assess the reasons as to why the operative diagnosis is associated with ADL limitations and not pain in patients undergoing revision THA.

## Abbreviations

THA: Total hip arthroplasty; BMI: Body mass index; ASA: American society of anesthesiologists; ICD-9: International classification of diseases- ninth revision; GEE: Generalized estimating equations.

## Competing interests

There are no financial conflicts related directly to this study. J.A.S. has received research and travel grants from Takeda and Savient; and consultant fees from URL pharmaceuticals, Savient, Takeda, Ardea, Regeneron, Allergan and Novartis. D.G.L. has received royalties/speaker fees from Zimmer, Orthosonic and Osteotech, has been a paid consultant and owns stock in Pipeline Biomedical and his institution has received research funds from DePuy, Stryker, Biomet and Zimmer.

## Authors’ contributions

JAS was responsible for study concept and design, modification of study design, review and interpretation of analyses, drafting of the manuscript and making revisions to the manuscript. DGL provided modifications of study design, review and interpretation of analyses and making revisions to the manuscript. Both authors read and approved the final manuscript.

## Pre-publication history

The pre-publication history for this paper can be accessed here:

http://www.biomedcentral.com/1471-2474/14/210/prepub

## Supplementary Material

Additional file 1 **Appendix 1.** Non-responder characteristics revision THA. **Appendix 2.** Univariate association of Diagnosis with limitation of each activity. Click here for file
