# Supplementary material for: Operative diagnosis for revision total hip arthroplasty is associated with patient-reported outcomes (PROs)
Source: BMC Musculoskelet Disord. 2013 Jul 17;14:210. doi: 10.1186/1471-2474-14-210 (PMC3722075; doi:10.1186/1471-2474-14-210)
Supplement: Additional file 1 — Appendix 1. Non-responder characteristics revision THA. Appendix 2. Univariate association of Diagnosis with limitation of each activity. [file 1471-2474-14-210-S1.docx]

**Additional Files**

**Additional File 1. Characteristics of Survey Responders for the 2- and 5-year cohorts compared to non-responders**

|  | **2-year*** |  | **5-year*** |  |
| --- | --- | --- | --- | --- |
| **Variable** | **Odds Ratio (95% CI)** | **p-value** | **Odds Ratio (95% CI)** | **p-value** |
| Gender |  |  |  |  |
| Female | 1.0 (ref) |  | 1.0 (ref) |  |
| Male | 1.0 (0.9,1.1) | 0.94 | 1.0 (0.8,1.1) | 0.68 |
| Age Category |  |  |  |  |
| < 60 | 1.0 (ref) |  | 1.0 (ref) |  |
| 61-70 | 1.0 (0.9,1.2) | 0.80 | 1.0 (0.8,1.2) | 0.78 |
| 71-80 | 1.1 (1.0,1.3) | 0.17 | 1.1 (0.9,1.4) | 0.38 |
| >80 | 0.9 (0.7,1.1) | 0.29 | 0.8 (0.5,1.1) | 0.13 |
| BMI Category |  |  |  |  |
| <25 | 1.0 (ref) |  | 1.0 (ref) |  |
| 25-29.9 | 1.0 (0.9,1.2) | 0.76 | 1.1 (0.9,1.4) | 0.20 |
| 30-34.9 | 0.9 (0.8,1.1) | 0.47 | 1.1 (0.8,1.3) | 0.66 |
| 35-39.9 | **0.7 (0.6,1.0)** | **0.04** | 0.8 (0.5,1.1) | 0.16 |
| >40 | 0.8 (0.5,1.1) | 0.18 | 1.0 (0.6,1.7) | 0.90 |
| ASA Score |  |  |  |  |
| 1-2 | 1.0 (ref) |  | 1.0 (ref) |  |
| 3-4 | **0.7 (0.6,0.8)** | **<0.01** | **0.6 (0.5,0.7)** | **<0.01** |
| Deyo-Charlson Index (5 point increase) | **0.6 (0.5,0.8)** | **<0.01** | **0.6 (0.4,0.9)** | **<0.01** |
| Income Category |  |  |  |  |
| >$45K | 1.0 (ref) |  | 1.0 (ref) |  |
| < $35K | 0.9 (0.7,1.1) | 0.26 | 0.8 (0.6,1.0) | 0.11 |
| >$35K-$45K | 0.9 (0.7,1.1) | 0.25 | 0.8 (0.6,1.1) | 0.13 |
|  |  |  |  |  |
| Distance Category |  |  |  |  |
| 0-100 miles | 1.0 (ref) |  | 1.0 (ref) |  |
| >100-500 miles | 1.1 (1.0,1.3) | 0.14 | 1.1 (0.9,1.4) | 0.18 |
| >500 miles or Non-US | 1.2 (1.0,1.5) | 0.08 | 1.1 (0.8,1.4) | 0.60 |
| Operative Diagnosis |  |  |  |  |
| Loosening/Wear or Osteolysis | 1.0 (ref) |  | 1.0 (ref) |  |
| Dislocation, Bone or Prosthesis Fracture, Instability, Non-Union | **0.4 (0.4,0.5)** | **<0.01** | **0.5 (0.4,0.7)** | **<0.01** |
| Failed Prior Arthroplasty with Components Removed or Infection | **0.6 (0.5,0.7)** | **<0.01** | **0.7 (0.6,1.0)** | **0.03** |

**Significant Odds ratios are in bold**

***Reference category is non-responders at the respective time (2-years or 5-years)**

**CI, confidence interval**

**Additional File** 2. Univariate association of Operative Diagnosis* with limitation of each activity

|  | **2-years** | | | **5-years** | | |
| --- | --- | --- | --- | --- | --- | --- |
|  | **OR** | **95% CI** | **p-value** | **OR** | **95% CI** | **p-value** |
| **Walking Limitations** |  |  |  |  |  |  |
| Dislocation, Bone or Prosthesis Fracture, Instability, Non-Union | **1.7** | **(1.3,2.0)** | **<0.01** | **1.5** | **(1.1,2.0)** | **<0.01** |
| Failed Prior Arthroplasty with Components Removed or Infection | **1.7** | **(1.3,2.2)** | **<0.01** | **1.6** | **(1.2,2.3)** | **<0.01** |
|  |  |  |  |  |  |  |
| **Stair Limitations** |  |  |  |  |  |  |
| Dislocation, Bone or Prosthesis Fracture, Instability, Non-Union | **2.1** | **(1.7,2.6)** | **<0.01** | **2.3** | **(1.7,3.0)** | **<0.01** |
| Failed Prior Arthroplasty with Components Removed or Infection | **1.8** | **(1.4,2.4)** | **<0.01** | 1.3 | (1.0,1.9) | 0.08 |
|  |  |  |  |  |  |  |
| **Socks/Shoes Limitations** |  |  |  |  |  |  |
| Dislocation, Bone or Prosthesis Fracture, Instability, Non-Union | 1.4 | (1.1,1.8) | <0.01 | 1.4 | (1.1,1.9) | 0.01 |
| Failed Prior Arthroplasty with Components Removed or Infection | 1.6 | (1.3,2.1) | <0.01 | 1.6 | (1.1,2.1) | <0.01 |
|  |  |  |  |  |  |  |
| **Pick up Objects Limitations** |  |  |  |  |  |  |
| Dislocation, Bone or Prosthesis Fracture, Instability, Non-Union | **1.5** | **(1.2,1.8)** | **<0.01** | **1.5** | **(1.1,2.0)** | **<0.01** |
| Failed Prior Arthroplasty with Components Removed or Infection | **1.5** | **(1.2,1.9)** | **<0.01** | 1.2 | (0.9,1.7) | 0.23 |
|  |  |  |  |  |  |  |
| **In/Out Car Limitations** |  |  |  |  |  |  |
| Dislocation, Bone or Prosthesis Fracture, Instability, Non-Union | **1.5** | **(1.2,1.9)** | **<0.01** | **1.5** | **(1.1,2.0)** | **<0.01** |
| Failed Prior Arthroplasty with Components Removed or Infection | **1.3** | **(1.0,1.7)** | **0.04** | 1.2 | (0.8,1.6) | 0.39 |
|  |  |  |  |  |  |  |
| **Rise from Chair Limitations** |  |  |  |  |  |  |
| Dislocation, Bone or Prosthesis Fracture, Instability, Non-Union | **1.7** | **(1.3,2.2)** | **<0.01** | **2.0** | **(1.4,2.7)** | **<0.01** |
| Failed Prior Arthroplasty with Components Removed or Infection | **1.7** | **(1.2,2.3)** | **<0.01** | **1.5** | **(1.0,2.3)** | **0.03** |
|  |  |  |  |  |  |  |
| **Sitting Limitations** |  |  |  |  |  |  |
| Dislocation, Bone or Prosthesis Fracture, Instability, Non-Union | **1.9** | **(1.0,3.7)** | **0.05** | 1.5 | (0.6,3.7) | 0.40 |
| Failed Prior Arthroplasty with Components Removed or Infection | 0.7 | (0.2,2.3) | 0.53 | 1.0 | (0.3,3.4) | 0.99 |

**Significant Odds ratios are in bold**

***Loosening, wear or osteolysis is the reference diagnosis category**
